# Isolation of ‘*Candidatus* Nitrosocosmicus franklandus’, a novel ureolytic soil archaeal ammonia oxidiser with tolerance to high ammonia concentration

**DOI:** 10.1093/femsec/fiw057

**Published:** 2016-03-13

**Authors:** Laura E. Lehtovirta-Morley, Jenna Ross, Linda Hink, Eva B. Weber, Cécile Gubry-Rangin, Cécile Thion, James I. Prosser, Graeme W. Nicol

**Affiliations:** Institute of Biological and Environmental Sciences, Cruickshank Building, St Machar Drive, University of Aberdeen, Aberdeen AB24 3UU, UK

**Keywords:** Thaumarchaeota, *Nitrososphaera* sister cluster, soil, ammonia inhibition, *Nitrosocosmicus*, nitrification

## Abstract

Studies of the distribution of ammonia oxidising archaea (AOA) and bacteria (AOB) suggest distinct ecological niches characterised by ammonia concentration and pH, arising through differences in substrate affinity and ammonia tolerance. AOA form five distinct phylogenetic clades, one of which, the ‘*Nitrososphaera* sister cluster’, has no cultivated isolate. A representative of this cluster, named ‘*Candidatus* Nitrosocosmicus franklandus’, was isolated from a pH 7.5 arable soil and we propose a new cluster name: *‘Nitrosocosmicus’*. While phylogenetic analysis of *amoA* genes indicates its association with the *Nitrososphaera* sister cluster, analysis of 16S rRNA genes provided no support for a relative branching that is consistent with a ‘sister cluster’, indicating placement within a lineage of the order *Nitrososphaerales*. ‘*Ca.* N. franklandus’ is capable of ureolytic growth and its tolerances to nitrite and ammonia are higher than in other AOA and similar to those of typical soil AOB. Similarity of other growth characteristics of ‘*Ca.* N. franklandus’ with those of typical soil AOB isolates reduces support for niche differentiation between soil AOA and AOB and suggests that AOA have a wider physiological diversity than previously suspected. In particular, the high ammonia tolerance of ‘*Ca.* N. franklandus’ suggests potential contributions to nitrification in fertilised soils.

## Abstract (precis)

This study describes ecophysiological characteristics of the first cultivated representative of a soil archaeal group performing an important biogeochemical process, ammonia oxidation, with significance for niche specialisation and archaeal phylogeny.

## INTRODUCTION

Soil nitrification, the sequential oxidation of ammonia to nitrite and nitrate, is generally limited by the activity of ammonia oxidisers (AO), which perform the first step in this process. Traditionally, ammonia oxidation was thought to be dominated by ammonia oxidising bacteria (AOB), but the cultivation of autotrophic ammonia oxidising archaea (AOA) from marine (Könneke *et al.*^2005^) and subsequently terrestrial (Lehtovirta-Morley *et al.*^2011^; Tourna *et al.*^2011^) environments led to a reassessment of the microbial ecology of soil nitrification. Potential differences in archaeal and bacterial physiology presented the possibility of niche differentiation between AOB and AOA. This is exemplified by the high affinity for ammonia of the marine isolate *Nitrosopumilus maritimus* (Martens-Habbena *et al.*^2009^), which explains the dominance of AOA in oligotrophic sea water, and by acidophilic growth of the soil isolates, *Nitrosotalea devanaterra* and *Nitrosotalea* sp. Nd2, which provides an explanation for nitrification in acid soils (Lehtovirta-Morley *et al.*^2011^, ^2014^). There is limited evidence for greater sensitivity of AOA to ammonia, with inhibition of growth at micromolar concentrations of ammonia, while AOB typically grow at millimolar concentrations (Koper *et al.*^2010^; Prosser and Nicol 2012). In addition, differences in effects of organic compounds have been reported within and between AOA and AOB (Lehtovirta-Morley *et al.*^2011^, ^2014^; Sayavedra-Soto and Arp 2011; Tourna *et al.*^2011^) and there is evidence for greater nitrite sensitivity in *N. maritimus* (presented in terms of nitrous acid in Table 1). Cell mass and specific cell activity are approximately one order of magnitude lower in cultivated soil AOA than soil AOB but maximum specific growth rates are similar (Prosser and Nicol 2012; Table 1).

**Table 1.** Characteristics of the ‘model’ AOA (*N. maritimus*) and AOB (*N. europaea*), six AOA soil isolates or enrichments and a range of soil AOB. Some data are not available for certain strains and cell yield and activity data for *Nitrosotalea* strains are not directly comparable when presented in terms of NH_3_. Cell yield and specific cell activity of C13 calculated using cell and *amoA* gene concentrations are labelled ^a^ and ^b^, respectively.

			Cell yield	Specific cell activity	Inhibitory	Inhibitory	
Organism	Size (μm)	*μ*_*max*_ (h^−1^)	(cells μM^−1^ NH_3_)	(fmol NO_2_^−^ cell^−1^ h^−1^)	NH_4_^+^ conc. (mM)	HNO_2_ conc. (μM)	References
*Nitrosopumilus maritimus* SCM1	0.17–0.22 × 0.5–0.9	0.033	5 × 10^4^	0.53	2	0.028	Könneke *et al*. (2005)
*Nitrososphaera viennensis* EN76	0.6–0.8 diam	0.024			20	0.79	Stieglmeier *et al*. (2014a)
*Nitrosoarchaeum koreensis* MY1	0.3–0.5 × 0.6 – 1.0		1.1 × 10^5^	2.5	20	0.50	Jung *et al*. (2011)
*Nitrosotenuis chungbukensis* MY2	0.2 × 0.7		3.4 × 10^5^		20	0.50	Jung *et al*. (2014)
*Nitrosotalea devanaterra* Nd1	0.33 × 0.89	0.011	4.5 × 10^5^	0.072	50	0.91–3.5	Lehtovirta-Morley *et al*. (2011)
*Nitrosotalea* sp. Nd2	0.2 × 0.7	0.025	4 × 10^5^	0.065		1.61–5.7	Lehtovirta-Morley *et al*. (2014)
*Nitrosocosmicus franklandus* (C13)	1.1 diam	0.024	^a^7.60 × 10^3^/ ^b^1.29 × 10^5^	^a^2.02 ^b^0.58	>100 mM	1.58	This study
*Nitrosomonas europaea* ATCC 19718	0.8–1.1 × 1.0–1.7	0.052–0.066	4.61–6.44 × 10^3^	11	>400	31.6	Prosser (1989); Hunik, Meijer and Tramper (1992); Koops *et al.* (2006)
Soil AOB (data collated from approximately 25 strains)	0.3–0.8 × 1.0–8.0	0.005–0.044	1.38–10.6 × 10^3^	4–23	7–50		Prosser (1989); Koops and Pommerening-Röser (2001); Koper *et al*. (2010); Prosser and Nicol (2012)

Characterisation of soil AOA and comparison with AOB are limited by difficulties in isolation of pure cultures, due to low specific growth rates of AO and their susceptibility to contamination by faster growing heterotrophs. Pure cultures of AOB have been available since their first isolation from soil by Frankland and Frankland (1890), although few strains have been subjected to detailed physiological analysis. In contrast, only nine AOA have been isolated to date: *N. maritimus* strains SCM1 (from a salt water aquarium) (Könneke *et al.*^2005^), NAO2 and NAO6 (from marine surface water) (Elling *et al.*^2015^), *N. devanaterra* Nd1 and *Nitrosotalea* sp. Nd2 (from acidic soils) (Lehtovirta-Morley *et al.*^2011^, ^2014^), *Nitrososphaera viennensis* EN76 (from garden soil) (Tourna *et al.*^2011^), *N. gargensis* Ga9.2 (from a hot spring) (Palatinszky *et al.*^2015^) and *Nitrosopumilus* strains PS0 and HCA1 (closely related to *N. maritimus* SCM1, isolated from coastal waters) (Qin *et al.*^2014^). Only three of these isolates are from soil, but two soil enrichments have also been characterised, *Nitrosoarchaeum koreensis* MY1 (Jung *et al*. 2011) and *Nitrosotenuis chungbukensis* MY2 (Jung *et al.*^2014^).

AOA and AOB use the enzyme ammonia monooxygenase (AMO) in the first step of ammonia oxidation, whose three subunits, A, B, C, are encoded by *amoA*, *amoB* and *amoC* genes, respectively. The *amoA* gene has been used extensively as a marker in diversity analyses, and initial studies showed that AOA are widely distributed in most environments (soil, marine waters and sediments, freshwater, geothermal springs) (e.g. Francis *et al.*^2005^; Reigstad *et al.*^2008^). In the soil, AOA typically outnumber AOB (e.g. Leininger *et al.*^2006^) and the relative abundance of phylogenetic groups within the AOA varies in different soils (Gubry-Rangin *et al.*^2011^).

A phylogenetic study (Pester *et al.*^2011^) of publically available archaeal *amoA* gene sequences placed all within one of five major clusters. Four of these clusters have characterised, cultured representatives (the *Nitrosopumilus*, *Nitrosotalea*, *Nitrosocaldus* and *Nitrososphaera* clusters). The fifth has a distinct, but specific association with the *Nitrososphaera* cluster, it was termed the ‘*Nitrososphaera* sister’ cluster (Pester *et al.*^2011^) and its phylogenetic divergence occurred early in thaumarchaeotal diversification (Gubry-Rangin *et al*. 2015). Sequences within this group are widely distributed in soil and other environments, but typically do not comprise the major proportion of *amoA* gene sequences in high throughput sequencing studies of soil (e.g. Gubry-Rangin *et al.*^2011^). Pester *et al.* (2011) found *amoA* gene sequences representative of this group in seven of nine soils sampled from four different continents, comprising up to 13% of all archaeal *amoA* gene sequences. In addition, a genome sequence of an unpublished soil enrichment culture has been deposited in GenBank (accession number CP012850.1) and has provisionally been named ‘*Candidatus* Nitrosocosmicus oleophilus’, with the proposed genus name describing ‘a widely distributed AO’ (Rhee, pers. comm.).

The aim of this study was to characterise a novel AOA belonging to the *Nitrososphaera* sister cluster (Pester *et al.*^2011^), isolated from a near-neutral pH agricultural soil, and to consider the implications for niche differentiation of AOA and AOB in soil.

## MATERIALS AND METHODS

### Culture enrichment and isolation

Sandy loam soil of pH 7.5 was sampled from an agricultural plot on the Scottish Rural College's Craibstone estate, Aberdeen (grid reference NJ872104). AO enrichment cultures were obtained by inoculation of 0.5 g soil in 50 ml ‘fresh water medium’ (FWM), containing NaCl (1 g l^−1^), MgCl_2_·6H_2_O (0.4 g l^−1^), CaCl_2_·2H_2_O (0.1 g l^−1^), KH_2_PO_4_ (0.2 g l^−1^), KCl (0.5 g l^−1^), NaHCO_3_ (0.168 g l^−1^), NH_4_Cl (0.027 g l^−1^) and 1 ml l^−1^ additions of the following solutions: modified non-chelated trace elements (Könneke *et al.*^2005^), Fe-NaEDTA (7.5 mM) (Tourna *et al.*^2011^) and 50 mg l^–1^ streptomycin sulphate. Medium was adjusted to pH 7.5 at room temperature and filter-sterilised through a bottle-top filter (0.22 μm, Merck Millipore, Watford) into 100-ml Duran bottles (Sigma-Aldrich, Gillingham, UK). Soil suspensions were initially incubated statically at 37°C in the dark, monitored for nitrite accumulation and subcultured (2% inoculum) when nitrite concentration increased exponentially. Nitrite and ammonium concentrations were determined colorimetrically using Griess reagent and indophenol method, respectively, as previously described (Lehtovirta-Morley *et al*. 2014). Reactions and absorbance measurements were performed in clear polystyrene 96-well microplates (Greiner Bio-One, Stonehouse, UK) and measured using an Infinite F50 Microplate Reader and Magellan reader control and data analysis software (Tecan, Männedorf, Switzerland).

The presence of bacteria in enrichment cultures was determined by spread-inoculating 1 ml of culture onto Tryptone Soy, Nutrient and LB Agar plates (Sigma-Aldrich, Gillingham, UK) at 5%, 10% and 25% of standard concentrations, respectively, and 1.5% w/v agar. Agar plates were incubated in the dark at 30°C or 40°C. In addition, culture purity was assessed by PCR amplification of bacterial and fungal SSU rRNA genes. Cells were pelleted from liquid culture before extracting DNA using a standard bead-beating protocol with SDS extraction buffer and phenol:chloroform:isoamyl alcohol (Lehtovirta-Morley *et al.*^2011^). PCR was performed using BIOTAQ polymerase and dNTP mix (Bioline, London, UK) according to manufacturer's instruction (see Table 2 for details of primers and PCR cycling conditions).

**Table 2.** Details of primers and PCR cycling conditions used in this study.

Target gene	Primer	Sequence (5^′^-3^′^)	Reference	Cycling conditions
Bacterial 16S rRNA	27f1492r	AGAGTTTGGATCMTGGCTCAGGYYACCTTGTTACGACTT	Lane (1991) Nicol *et al.* (2008)	95°C 5 min; 10 cycles of 94°C 30 s, 55°C 30 s, 72°C 2 min; 25 cycles of 92°C 30 s, 55°C 30 s, 72°C 2 min; 72°C 5 min
Fungal 18S rRNA	Fung5fFF390r	GGGAACCAGGACTTTTACGAGGTCTCGTTCGTTATCG	Smit *et al.* (1999) Vainio and Hantula (2000)	95°C 5 min; 10 cycles of 94°C 30 s, 55°C 30 s, 72°C 50 s; 25 cycles of 92°C 30 s, 55°C 30 s, 72°C 50 s; 72°C 5 min
Archaeal 16S rRNA	A109F 1492r	ACKGCTCAGTAACACGT GYYACCTTGTTACGACTT	Grosskopf, Janssen and Liesack (1998) Nicol *et al.* (2008)	95°C 5 min; 10 cycles of 94°C 30 s, 55°C 30 s, 72°C 2 min; 25 cycles of 92°C 30 s, 55°C 30 s, 72°C 2 min; 72°C 5 min
Thaumarchaeal *amoA*	CrenamoA23fCrenamoA616r	ATGGTCTGGCTWAGACGGCCATCCATCTGTATGTCCA	Tourna *et al.* (2008) Tourna *et al.* (2008)	95°C 5 min; 10 cycles of 94°C 30 s, 55°C 30 s, 72°C 1 min; 25 cycles of 92°C 30 s, 55°C 30 s, 72°C 1 min; 72°C 5 min
*Nitrosotalea* sp. Nd2 *amoA* gene cluster	Nd2F1Nd2R2	GATACTTGCAGTGATACCTACCCCAGATATTCTTGTTTCAACAGAGG	This studyThis study	95°C 5 min; 35 cycles of 94°C 30 s, 52°C 30 s, 72°C 4 min; 72°C 10 min

To eliminate bacteria from enrichment cultures, their antibiotic sensitivity was determined by spread-inoculating 0.5 ml of an exponentially growing enrichment on FWM agar plates containing antibiotic discs and incubating at 40°C in the dark. Antibiotic discs were prepared by permeating sterile paper with 50 mg l^−1^ stock solutions (diluted in H_2_O) of ampicillin, carbenicillin, gentamycin, kanamycin, clindamycin, penicillin or rifampicin and dried in the dark at ambient temperature (Lehtovirta-Morley *et al.*^2014^). Antibiotics inhibiting bacterial contaminants were then added to FWM at a final concentration of 50 mg l^−1^, in an attempt to purify the enrichment culture.

### Culture maintenance and preservation

The purified archaeal strain, initially termed C13, was routinely cultured in FWM as described above but with the addition of 1 ml l^−1^ of the following solutions: vitamin solution (excluding C_5_H_14_NO^+^) (Lehtovirta-Morley *et al.*^2011^), Se-We solution (Widdel and Bak 1992) and 10 ml l^−1^ of HEPES buffer (1 M HEPES, 0.6 M NaOH), with or without clindamycin (50 mg l^−1^) and ampicillin (100 mg l^−1^). All solutions were added prior to filter sterilisation.

C13 was cryopreserved and stored at –80°C using dimethylsulfoxide (DMSO) (VWR, Lutterworth, UK) as a cryoprotectant. DMSO was diluted to a concentration of 14% (v/v) using FWM and filter-sterilised (0.22-μm filter) before the addition of 0.5 ml DMSO solution to 0.5 ml of an exponentially growing culture in a sterile 1.5 ml microcentrifuge tube. Tubes were agitated for 5 min at 25°C in the dark to allow the DMSO to penetrate the cells before storage at –80°C. To resuscitate cells, samples were thawed at 25°C and immediately centrifuged at 4°C at 1200 × *g* for 10 min to minimise the cytotoxic effects of DMSO. Supernatant was then removed, pelleted cells were resuspended in 1 ml sterile FWM and centrifuged and washed three times, to remove residual DMSO, before final resuspension in 10 ml FWM. Growth of cells preserved for 3 weeks was detectable after resuscitation for 4 days.

### Physiological characterisation

Unless otherwise stated, all physiological characteristics were determined during batch cultivation, with static incubation of triplicate cultures in FWM in the dark at 40°C as described above for enrichment cultures. The effects of ammonium concentration were determined in FWM supplemented with 0, 1, 2, 5, 10, 20, 50 or 100 mM NH_4_Cl and inhibition by nitrite concentration by supplementation with 0, 1, 2, 5, 10, 20 or 50 mM NaNO_2_. Ureolytic activity was investigated in ammonia-free FWM supplemented with 1 mM urea. Growth was also determined during incubation at temperatures ranging from 15°C to 60°C (5°C intervals). The effect of initial pH value in the range of 5–9 (0.5 pH intervals) was investigated in unbuffered medium containing a reduced concentration of 100 μm NH_4_Cl, to minimise the decrease in pH decline due to ammonia oxidation, and in medium buffered with 5 mM MES (2-(*N*-morpholino)ethanesulfonicacid) or 10 mM HEPES (4-(2-hydroxyethyl)-1-piperazineethanesulfonicacid). As MES and HEPES have p*K*_*a*_ values of 5.91 and 7.31, respectively, at 40°C, neither possessed the buffering capacity to fulfil the full range of pH tested in this study, and MES buffer was used at pH 5.5–7 and HEPES buffer at pH 7–8.5.

### Cell counts and microscopy

Cells were enumerated microscopically in 1 ml samples of liquid culture, fixed with 5% formaldehyde (final concentration; w/v) and stored at 4°C. Each sample was repeatedly passed through a syringe and needle (0.4 mm diameter) to disperse aggregated cells. Cells were then diluted and stained with 2 μg DAPI (4, 6-diamidino-2-phenylindole), incubated for 10–15 min at 4°C in the dark, filtered onto a black 0.2-μm polycarbonate membrane and washed with PBS. Dried filters were placed on a glass slide mounted with an antifadent (Citifluor AF2, Citifluor Ltd, Leicester, UK) and counted under oil immersion using an Olympus BH-2 microscope with a U-MWU2 fluorescence light source (Olympus, Southend-on-Sea, UK) at ×1000 magnification. For scanning electron microscopy, cells were fixed and gold-sputtered as previously described (Lehtovirta-Morley *et al.*^2014^) and images taken using an Evo MA 10 scanning electron microscope (Carl Zeiss, London, UK).

### Abundance of *amoA* genes

Growth of strain C13 was also determined by quantification of *amoA* genes. DNA was extracted as described above, except that 20 μg glycogen was added as co-precipitant during DNA precipitation. qPCR amplification was performed in a BioRad MyIQ Single-Color Real-Time PCR Detection System (Hertfordshire, UK) using amplification-conditions and cycling-parameters described in Thion and Prosser (2014), except that 0.6 μm of each primer was used. A PCR product containing the *amo* gene cluster of *Nitrosotalea* sp. Nd2 (see Table 2 for details of primers and PCR cycling conditions) was used as a standard at dilutions giving 10^2^–10^8^*amoA* genes. The amplification efficiency was 97% and the *r*^2^ value 0.99. Positive amplification was confirmed by melting curve analysis and agarose gel electrophoresis.

### DOPE-FISH analysis

Purity of strain C13 was also determined by DOPE-FISH (double labelling of oligonucleotide probes for fluorescence *in situ* hybridisation) using archaeal ARCH915 probe (Stahl and Amann 1991) labelled with Cy3 and bacterial EUB338 probe (Daims *et al.*^1999^) labelled with fluorescein. One ml of stationary phase culture was washed twice with PBS, concentrated in 100 μl of PBS and fixed with 96% ethanol (1:1 v/v). Before applying the cells to a teflon-covered microscope slide, cells in the fixed culture were dispersed by passing through a syringe and needle as described above. After dehydrating the cells on a microscope slide, 1 μl of each doubly labelled FISH probe (50 ng μl^−1^) was added to 10 μl of hybridisation buffer using 30% stringency (0.9 M NaCl, 20 mM Tris-HCl, 30% (v/v) formamide and 0.01% SDS) (Stoecker *et al.*^2010^). Cells were incubated at 46°C for 4 h in the dark, after which the microscope slide was transferred to the pre-warmed washing buffer (112 mM NaCl, 20 mM Tris-HCl and 5 mM EDTA) and incubated for 15 min at 48°C. Cells were then resuspended in 100 μl of pre-warmed washing buffer and incubated at 48°C for 20 min. Finally, the microscope slide was dried and counter- stained with DAPI in an antifadent oil (Citifluor AF1, Citifluor Ltd, Leicester, UK) and samples were visualised using a Zeiss Imager M2 fluorescent microscope (Carl Zeiss, London, UK).

### Phylogenetic analysis

PCR was performed with primer sets targeting the archaeal *amoA* gene (CrenamoA23f/CrenamoA616r) and 16S rRNA gene (A109f/1492r) (Table 2). PCR products were purified using a Macherey–Nagel NucleoSpin PCR Clean-up Kit (Fisher Scientific, Loughborough, UK) and sequenced, with assembled 16S rRNA and *amoA* gene sequences deposited in GenBank with accession numbers KU290365 and KU290366, respectively. Sequences were aligned with those from all previously cultivated AOA and a selection of environmental clones using the ClustalW function within the BioEdit software (Hall 1999) before removing regions of ambiguous alignment. Phylogenetic analyses were performed using maximum-likelihood with General Time Reversible-correction (PhyML; Guindon and Gascuel 2003), Bayesian (MrBayes (Ronquist *et al.*^2012^), parsimony (MEGA5 (Tamura *et al.*^2011^)) and Tamura's 3-parameter pairwise distance analysis (MEGA5) for 16S rRNA gene analysis, and Jones-Taylor-Thornton (JTT)-corrected maximum-likelihood (PhyML), Bayesian (MrBayes), parsimony (MEGA5) and JTT-corrected pairwise distance (MEGA5) analyses of inferred translated amino acid sequences for *amoA* genes. Where appropriate, analyses used estimated variable sites only with gamma-distributed site variation and bootstrap support was calculated 1000 times for ML, parsimony, distance analyses, with Bayesian analysis performed with 100 000 iterations, sampling frequencies of 10, with an average standard deviation <0.01.

### Statistical analysis

Maximum specific growth rate, *μ*_*max*_, was calculated as the gradient of semi-logarithmic plots of nitrite, cell or *amoA* gene concentration vs time during exponential growth. The effect of treatments on *μ*_*max*_ was analysed by one-way ANOVA and linear regression analysis was performed on *μ*_*max*_ using Excel. Cell activity was determined for each replicate culture by minimising the sum of squares of differences between nitrite concentrations determined experimentally and those predicted by assumption of constant specific cell activity during exponential growth using the Solver routine in Excel, with *μ*_*max*_ and initial cell concentration or *amoA* gene concentration determined experimentally for each replicate culture.

## RESULTS

### Strain isolation

Enrichment cultures containing Thaumarchaeota, identified by amplification and sequencing of *amoA* genes, were obtained by inoculation of inorganic (FWM) medium containing 0.5 mM NH_4_^+^ with a sandy loam soil, pH 7.5. One culture containing a single *amoA* gene clonal population (designated C13) was selected for purification. Growth of heterotrophic bacteria was observed on Nutrient, Tryptone Soy and LB agar, but was eliminated after seven successive subcultures in FWM containing 500 mg l^−1^ kanamycin. Bacterial contamination was, however, detected by PCR amplification of 16S rRNA genes and by growth on solid FWM.

**Figure 1.**
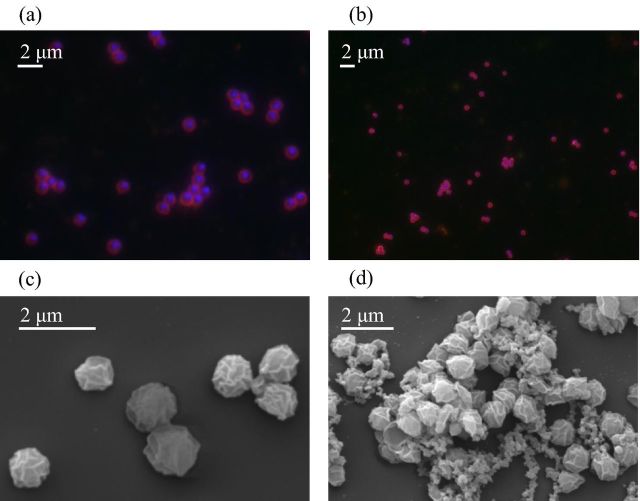
Typical DOPE-FISH images of stationary-phase C13 culture (**a**, **b**) using archaeal ARCH915 probe (red), bacterial EUB338 probe (green) and counterstained with DNA DAPI stain (blue). Scanning electron micrographs of individual cells (**c**) and aggregates (**d**) of archaeal ammonia oxidiser strain C13.

Heterotrophic contamination was reduced further by supplementation of medium with one of five antibiotics (ampicillin, carbenicillin, gentamycin, clindamycin and rifampicin) to which bacterial contaminants were sensitive. Nitrite was produced in medium containing each antibiotic except gentamycin, but was not detectable in subsequent subcultures, presumably because of a requirement for compounds produced by co-cultured bacteria. Growth was restored by the addition of spent (filter-sterilised) growth medium (1:10 ratio) and the addition of clindamycin and ampicillin. After a further eight subcultures with addition of spent medium and antibiotics, bacterial growth was not detected on FWM agar plates incubated for two weeks. Subsequent growth occurred in the absence of spent medium with no detectable amplification of bacterial 16S rRNA genes. The purity of strain C13 was also confirmed by DOPE-FISH which revealed the presence of archaeal cells, but never bacteria (Fig. 1a, b).

### Physiological characteristics

**Figure 2.**
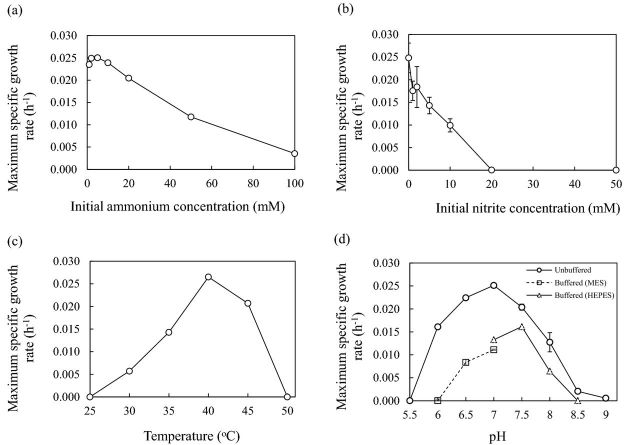
The influence of (**a**) initial ammonium concentration, (**b**) initial nitrite concentration, (**c**) temperature and (**d**) pH on maximum specific growth rate of archaeal ammonia oxidiser isolate in batch culture. Growth in (d) was in unbuffered FWM or FWM buffered with MES or HEPES. Error bars are standard errors of means from triplicate cultures but are often smaller than the symbol size.

The influence of ammonia concentration on growth was determined by cultivation of strain C13 in medium containing 0–100 mM NH_4_^+^ (Fig. 2a). *μ*_*max*_ was greatest (0.025 [s.e. 0.0002] h^−1^) at 5 mM, and lower at 1 mM. *μ*_*max*_ decreased with increasing ammonium concentration, but growth was possible at the highest concentration tested, 100 mM, at 0.0035 [s.e. 0.0002] h^−1^. Nitrite was tolerated at initial concentrations of 0–10 mM NO_2_^−^ (*p* = 0.13), but growth was completely inhibited by 20 mM and 50 mM NO_2_^−^, with no detectable increase in nitrite concentration after incubation for 21 days (Fig. 2b).

Strain C13 is mesophilic and grew at 30°C–45°C (Fig. 2c), with no detectable increase in cell abundance or nitrite concentration outside this temperature range after incubation for 21 days. Growth was optimal at 40°C, with a *μ*_*max*_ of 0.027 [s.e. 0.00006] h^−1^. The isolate is neutrophilic, growing in the pH range 6–8.5 (Fig. 2d), and no detectable increase in cell abundance or nitrite concentration occurred outside this pH range during incubation for 21 days. Growth in unbuffered FWM was optimal at pH 7, with a maximum specific growth rate of 0.025 [s.e. 0.0002] h^−1^ (Fig. 2d), but pH in this medium varied during growth, increasing to 7.5 after incubation for 4 days, and subsequently decreasing to 7.3 when ammonium was completely utilised. Growth in FWM buffered by addition of MES or HEPES was optimal at pH 7.5 (Fig. 2d), but the *μ*_*max*_ was lower than in unbuffered medium, at 0.016 [s.e. 0.00006] h^−1^, presumably due to inhibition by buffer.

**Figure 3.**
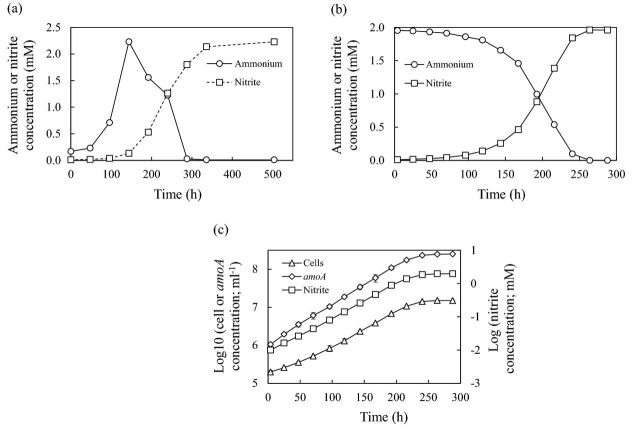
(**a**) Changes in ammonium and nitrite concentration during growth of strain C13 during batch growth on FWM with urea as nitrogen source; (**b**) changes in ammonium and nitrite concentrations and (**c**) semilogarithmic plots of nitrite (□), cell (▵) and *amoA* (◊) concentrations during growth on FWM with 2 mM ammonium chloride. Error bars are standard errors of means from triplicate cultures but are often smaller than the symbol size.

C13 is ureolytic and grew on medium containing 1 mM urea as the sole source of ammonia (Fig. 3a) with a *μ*_*max*_ of 0.025 s.e. 0.0005 h^−1^. Ammonia concentration increased from 65 μm at day 0 (carried over in inoculum) to >2 mM during incubation for 6 days and was stoichiometrically converted to nitrite (Fig. 3b). *μ*_*max*_ estimated as specific rates of increase in nitrite, cell and *amoA* concentration differed slightly (Fig. 3c, Table 3), although differences between *μ*_*max*_ values are statistically significant (t-test, *p* > 0.05). *amoA* gene concentrations were approximately one order of magnitude greater than cell concentrations, and cell activities and cell yields calculated using both sets of data are presented in Table 3.

**Table 3.** Growth characteristics of strain C13 during batch growth at 40°C in HEPES-buffered FWM at pH 7.5 with initial ammonia concentration of 2 mM and determined on the basis of changes in concentrations of nitrite, cells and *amoA* genes. Means and standard errors are calculated from triplicate cultures.

Basis for calculations	*μ*_*max*_ (h^−1^)	Specific cell activity (fmol NO_2_^−^ cell^−1^ h^−1^)	Cell yield (cells μM^−1^ NH_3_)
Nitrite concentration	0.0237 (s.e. 0.0001)		
Cell concentration	0.0248 (s.e. 0.0011)	2.02 (s.e. 0.19)	7.60 × 10^3^ (s.e. 0.19 × 10^3^)
*amoA* concentration	0.0239 (s.e. 0.0004)	0.58 (s.e. 0.17)	1.29 × 10^5^ (s.e. 0.097 × 10^5^)

### Morphology

Cells are irregularly shaped cocci with a diameter of 0.96 μm (standard error = 0.021 μm, range = 0.43–1.59 μm, *n =* 66) (Fig. 1c). Cells frequently occurred as chains of four cells or in irregular clusters, often with substantial extracellular material that was also observed detached from cells. These irregularly shaped aggregates varied in diameter from 7 to 15 μm (Fig. 1d).

### Phylogenetic analyses

**Figure 4.**
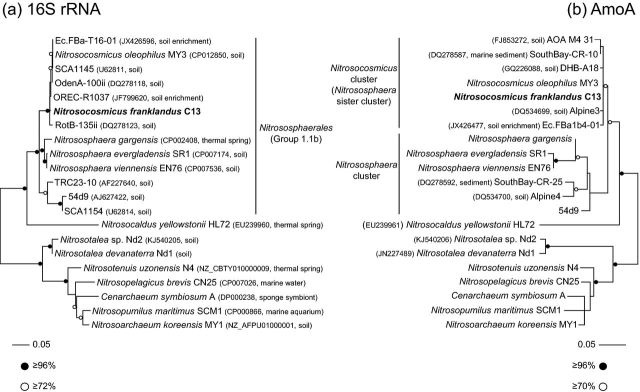
Maximum-likelihood phylogenetic analysis of (**a**) 16S rRNA genes and (**b**) derived AmoA protein sequences of isolated archaeon strain C13 with sequences from other cultivated organisms, metagenomes or cloned environmental sequences. Clade names are as described by Stieglmeier *et al.* (2014a) (16S rRNA) or Pester *et al.* (2011) (AmoA). Circles describing support represent the most conservative value from four methods used (bootstrap (ML, distance, parsimony) or posterior probabilities (Bayesian)) and the scale bar represents 0.05 changes per nucleotide or amino acid position. Accession number and environmental source are given in parentheses. If an accession number describes a genome or genomic fragment containing both a 16S rRNA and *amoA* gene, details are provided in the 16 rRNA tree only.

Phylogenetic analysis of 16S rRNA and *amoA* genes placed C13 within a well-supported, monophyletic lineage associated with organisms found in enrichment cultures and cloned sequences from various soil-based studies, with 16S rRNA and *amoA* genes sharing 99% and 92% identity, respectively, with those from the unpublished genome of ‘*Ca.* Nitrosocosmicus oleophilus’ and an enrichment culture obtained from arctic soil (Alves *et al.*^2013^). It was however, distinct from all cultured *Nitrososphaera* strains, with closest 16S rRNA similarity to *N. viennensis* at 95% identity (Fig. 4). While analysis of both genes shows a clear phylogenetic association with *Nitrososphaera*-like organisms, differences were observed when associating strain C13 (and closely related organisms) within a separate, sister cluster to the *Nitrososphaera*. Analysis of derived AmoA protein sequences demonstrated that C13 was placed in the clade designated by Pester *et al.* (2011) as the *Nitrososphaera* sister cluster (sharing 91%–99% identity with the 14 sequences analysed in that study), and distinct from those designated as belonging to the *Nitrososphaera* cluster. We thus propose the new clade delineation ‘*Nitrosocosmicus’* cluster for these sequences. However, analysis of 16S rRNA gene sequences did not provide support for a separate lineage affiliation, with C13 being placed within a lineage that is associated with other members of the *Nitrososphaerales* (Stieglmeier *et al.*2014a).

## DISCUSSION

The thaumarchaeotal strain C13 is a mesophilic, neutrophilic autotrophic archaeal AO that grows within the temperature range 30°C–45°C and pH range 6–8.5. In these respects, it is similar to the soil AOA *N. viennensis* (28°C–45°C, pH 6–8.5) (Stieglmeier *et al.*2014b) and *N. chungbukensis* MY2 (25°C–40°C, pH 6.5–8) (Jung *et al.*^2014^). It also utilises urea as a source of ammonia, in common with several other members of the ‘*Nitrososphaera*’ lineage that possess genes encoding urease and urea transporters and *N. viennensis* and *N. gargensis* that can grow on urea (Tourna *et al.*^2011^; Spang *et al.*^2012^; Zhalnina *et al.*^2014^).

Cells of strain C13 are larger than those of *N. viennensis* (0.6–0.8 μm diam) (Stieglmeier *et al.*2014a) and other soil AOA isolates, with an estimated cell volume of 0.7 μm^3^. Cells were associated with extracellular polymeric material that led to aggregate formation and may facilitate biofilm formation, which has been shown to protect AOB from deleterious effects of low pH and nitrification inhibitors (de Boer *et al.*^1991^; Powell and Prosser 1991; Allison and Prosser 1993). *μ*_*max*_ values were within the range found for other neutrophilic soil AOA (Table 1). Cell concentration was approximately one order of magnitude lower than *amoA* gene concentration, and similar differences have been reported in *N. maritimus* (Nakagawa and Stahl 2013). Cell concentration may be underestimated by incomplete dispersion of cells in aggregates, while *amoA* gene concentration may be overestimated by extracellular DNA following cell lysis. Cell yield and activity estimated using *amoA* gene concentrations are more consistent with those for other soil AOA and with *N. koreensis* MY1, respectively.

Strain C13 was more tolerant to ammonia than other soil AOA (Table 1), although the mechanisms for ammonia tolerance are unknown, and growth was possible at 100 mM NH_4_^+^, at which *μ*_*max*_ was reduced to approximately 10% of that at optimal ammonium concentration. Strain C13 was also more tolerant to inhibition by nitrite than other neutrophilic soil AOA and grew at initial concentrations up to 20 mM NO_2_^−^. At pH 7.5, this is equivalent to 1.58 μm HNO_2_, which is believed to cause inhibition, and nitrous acid tolerance of strain C13 is therefore similar to that of *N. devanaterra* and *Nitrosotalea* sp. Nd2, greater than that of other cultivated soil AOA and one order of magnitude greater and lower than *N. maritimus* and *Nitrosomonas europaea*, respectively (Table 1).

Comparisons of the ‘model’ AOA and AOB, *N. maritimus* and *N. europaea* ATCC 19718, have led to the view that AOA are smaller, have lower growth rates and specific cell activity and lower tolerance to ammonia and nitrite than AOB; these differences have further been suggested to drive niche differentiation between AOA and AOB. While these distinctions are evident for the model AOA and AOB (Table 1), and exist across the breadth of AOA and AOB, they are less marked when considering only soil isolates (Table 1). Soil AOA and AOB have a similar range for *μ*_*max*_ and are therefore likely to compete equally for ammonia when it is not growth-limiting. AOA are generally smaller and consequently have lower specific cell activity and greater cell yield than AOB, but this, in itself, provides no obvious competitive advantage. However, strain C13 extends the range of ammonia tolerance beyond that of characterised cultivated soil AOB (Prosser 1989; Koper *et al.*^2010^), reducing support for selective inhibition of AOA at high ammonia concentration, and suggesting a potential role for AOA in nitrification in fertilised soils. Care is therefore required when using information from a limited number of isolates to explain ecological phenomena and the differences between physiological characteristics of *Nitrosotalea* isolates (Lehtovirta-Morley *et al.*^2014^) highlight physiological diversity between strains that are very closely related in terms of 16S rRNA gene sequences. In addition, cultivation conditions will select strains that may not be representative of dominant and active members of natural communities and adaptation and selection will increase with continued cultivation. In this respect, C13 did not grow at 25°C and growth was optimal at 40°C, which is not typical of its environmental origin.

Phylogenetic analysis of predicted AmoA amino acid sequences places strain C13 in the *Nitrososphaera* sister cluster (Pester *et al.*^2011^), a lineage that is closely related to, but distinct from, the *Nitrososphaera* cluster. However, the same relative branching order was not resolved in 16S rRNA gene phylogenies, with strain C13 belonging to clade placed within, and not basal to, the order *Nitrososphaerales*, a lineage that is generally referred to as ‘Group 1.1b’ in environmental surveys (e.g. Nicol *et al.*^2008^). These contrasting topologies are consistent with a recent detailed comparison by Vico Oton *et al.* (2015) of thaumarchaeal 16S rRNA (clusters O and M) and *amoA* (C13_1 and C13_2) gene phylogenies. However, as strain C13 shares only 94%–95% 16S rRNA gene identity with other cultivated *Nitrososphaera* representatives, C13 probably represents a novel genus within the *Nitrososphaerales*.

This paper therefore reports the discovery of an AO that grows at neutral pH, but whose growth and activity are similar to those of soil AOB enrichments and isolates at neutral pH. Phylogenetic analysis of 16S rRNA and *amoA* genes places the organism within the *Nitrososphaerales* but distinct from previously isolated representatives of this order. We propose the following candidate status:

‘Nitrosocosmicus franklandus’ gen. et sp. nov.

Etymology. nitrosus (Latin masculine adjective): nitrous; cosmicus (Latin masculine adjective): cosmopolitan; Frankland (English noun): after Percy and Grace Faraday Frankland. The name reflects its ability to oxidise ammonia to nitrite, its widespread distribution in the environment, and provides a dedication to the authors of the first publication to describe isolation of organisms associated with ammonia oxidising activity (Frankland and Frankland 1890).

Source. Agricultural soil of pH 7.5, Craibstone, Aberdeen, Scotland, UK.

Description. A chemolithoautotrophic AO of the kingdom *Thaumarchaeota*, appearing as irregular cocci with a diameter 1.1 μm.

In accordance with other members of *Nitrososphaerales*, *N. franklandus* is an ammonia oxidising archaeon, isolated from a terrestrial environment, demonstrating the characteristic irregular coccoid cell morphology associated with this order (Stieglmeier *et al.*2014a). As with *N. viennensis*, it is able to grow on urea but it can tolerate higher ammonium concentrations, equivalent to those tolerated by oligotrophic bacterial AOs (Tourna *et al.*^2011^). *Nitrosocosmicus franklandus* represents an ecologically relevant strain of Thaumarchaeota with the potential to compete successfully with ammonium oxidising bacteria in fertilised soils where ammonium concentrations are high. The discovery of *N. franklandus* reduces support for niche differentiation between AOA and AOB in soil, with the possible exception in acid soils, and suggests that physiological diversity within each group may be more important in determining the composition of natural communities.
